# Editorial: Towards an understanding of tinnitus heterogeneity, volume II

**DOI:** 10.3389/fnagi.2024.1376600

**Published:** 2024-02-12

**Authors:** Christopher R. Cederroth, Tobias Kleinjung, Berthold Langguth, Arnaud Noreña, Patrick Neff, Birgit Mazurek, Pim Van Dijk, Winfried Schlee

**Affiliations:** ^1^Department of Physiology and Pharmacology, Karolinska Institutet, Stockholm, Sweden; ^2^Translational Hearing Research, Tübingen Hearing Research Center, Department of Otolaryngology, Head, and Neck Surgery, University of Tübingen, Tübingen, Germany; ^3^Department of Otorhinolaryngology, Head, and Neck Surgery, University Hospital Zurich, University of Zurich, Zurich, Switzerland; ^4^Department of Psychiatry and Psychotherapy, University of Regensburg, Regensburg, Germany; ^5^Laboratoire de Neurosciences Cognitives, UMR 7291, Centre National de la Recherche Scientifique, Aix-Marseille University, Marseille, France; ^6^Tinnitus Center, Charité-Universitätsmedizin Berlin, Corporate Member of Freie Universität Berlin and Humboldt-Universität zu Berlin, Berlin, Germany; ^7^Department of Otorhinolaryngology-Head and Neck Surgery, University of Groningen, University Medical Centre Groningen, Groningen, Netherlands; ^8^Eastern Switzerland University of Applied Sciences, St. Gallen, Switzerland

**Keywords:** tinnitus, heterogeneity, psychology, neurology, treatment, cochlear implant

In 2016, we were launching the first Research Topic on the heterogeneity of tinnitus having in aim the challenge for developing a uniformly effective treatment for all tinnitus patients. Toward the end of 2017, 79 published peer-reviewed articles composed the first issue of the Research Topic (Cederroth et al.), covering an outstanding view on the current research status in the field including tinnitus profiles, comorbidities, psychological distress, and therapy. This second issue on understanding of tinnitus heterogeneity presents a continued view on tinnitus, its fundamental understanding and its treatment, with an emphasis on auditory, psychoacoustics, psychology and neuroimaging approaches.

## 1 Statistics on this Research Topics

The Research Topic was open between December 2021 and December 2022. It received 33 manuscripts by 231 contributors of which 26 were accepted after peer-review. At the moment of submitting this editorial, the Research Topic achieved >109,000 article views and >19,000 downloads. Notably, a randomized single-blind controlled trial on a polytherapeutic tinnitus treatment app by Searchfield and Sanders reached a high social media visibility, with an Altmetric score of 614.

## 2 Overview of this Research Topic

This second issue on Tinnitus Heterogeneity begins with two pieces (one opinion and one hypothesis) on the psychological struggle on the perception of time in patients with chronic tinnitus (Dauman) and the potential mechanisms underlying the improved cognitive performance in these patients (Schilling and Krauss). The first chapter comprises a series of one systematic review on the auditory brainstem responses and the correlation of the short and middle latencies with tinnitus, and two reviews focusing on the involvement of stress in the development of tinnitus and the management of tinnitus in individuals with severe-to-profound hearing loss. The second chapter, composed of the single computational modeling article, evaluates how to improve speech recognition by adding intrinsic noise, something that may be at the origin of tinnitus. Three articles on animal-based work, covering the behavioral assessment of tinnitus, and the contributions of salicylate or noise exposure in stochastic resonance and the hyperactivity of cortical neurons, complete it. The final chapter is a dense coverage of human work on auditory, psychological and neuroimaging – some of these revealing the increasing contribution of inflammatory processes on tinnitus.

## 3 Chapter 1: reviews

This Research Topic also includes three reviews. In one review Patil et al. discuss the association between stress, emotional states, and the development of tinnitus. They summarize the literature on alterations in the hypothalamic–pituitary–adrenal (HPA) axis in tinnitus patients and integrate these findings with epidemiologic data supporting the role of stress as a risk factor for tinnitus. Jacxsens et al. performed a meta-analysis of brainstem evoked auditory potentials in tinnitus. They demonstrated delayed short-latency AEPs (auditory evoked potentials) in tinnitus patients. These results suggest tinnitus-related alterations at brainstem level. They speculate that the prolongation of ABR (auditory brainstem response) latencies may be related to high-frequency sensorineural hearing loss, cochlear synaptopathy, or somatosensory tinnitus generators. No clear conclusions could be drawn regarding middle-latency AEPs, which represent the subcortical level of the auditory pathway. Alzahrani et al. reviewed the existing literature regarding the experience and management of tinnitus in adults who have severe-to-profound hearing loss. They concluded that the available literature focuses primarily on cochlear implant care for severe-to-profound hearing loss, while empirical studies seeking to understand the nature of the tinnitus experience of people with no or little residual exposure to external sounds are largely missing.

## 4 Chapter 2: computational and animal studies

Noise trauma or salicylate administration are well-known to induce neural hyperactivity in the auditory centers. The cellular mechanisms of these neural changes, which have been hypothesized to be neural corelates of tinnitus, are unknown. In animal research, it is appropriate to develop a behavioral correlate of the presence of tinnitus, so that the neural correlates of tinnitus can be understood with good confidence. In this context, the article of Fabrizio-Stover et al. compared two behavioral methods to reveal the presence of tinnitus, an active avoidance paradigm (AA) and the gap-induced pre-pulse inhibition of acoustic startle (GPIAS). Only 10% of animals (mice) were positive to both tests. Interestingly, the authors used a neuronal marker to differentiate the two methods and showed that the spontaneous activity was increased only in animals positive to AA. The authors concluded that AA may be more reliable than GPIAS. Lanaia et al. tested the correlation between the putative presence of tinnitus induced by salicylate administration assessed with the GPIAS and the hearing threshold shift derived from ABRs. There was no correlation between these two variables. The authors concluded that salicylate-induced tinnitus is likely not the result of a neural mechanism involving stochastic resonance. The study of Nogueira et al. addresses the cellular mechanisms after a noise-induced hearing loss. The authors demonstrated that Martonotti cells in layer 5 show a more hyperpolarized resting membrane potential in slices from noise-exposed mice compared to control. As the L5 comprises neurons that send feedback to other areas, noise-exposed changes may alter levels of activity of the descending and contralateral auditory system.

## 5 Chapter 3: human

### 5.1 Randomized controlled trial

One randomized controlled trial was submitted to this Research Topic. In this trial, Searchfield and Sanders compared two digital health interventions on 98 enrolled participants. A noise generator smartphone app was used as active control and tested against a digital polytherapeutic approach (smartphone app plus bone conduction headphones plus neck pillow speakers), which provided three different treatment methods: counseling, passive, and active listening tasks. The study was carried out during the Corona pandemic, and the drop-out rate was 38% - much higher than the 5% drop-out rate anticipated for the power calculation. This might have influenced the results, which showed no significant difference between the two treatment arms. The reported Cohen's *d* effect size was 1.01 for the polytherapeutic treatment and 0.57 for the active control condition with self-administered sound stimulation.

### 5.2 Psychological, auditory, psychoacoustics

In this section, effects of different somatic and psychological comorbidities, including hearing impairment, on the expression of tinnitus distress are examined. Very recently, Jarach et al. used a survey in northern Italy to address the question of whether tinnitus and other hearing impairments increased in the context of the COVID-19 pandemic as a strong social stressor, something that could not be confirmed. Tinnitus incidence decreased in 2020 compared with previous years, and distress was described as remaining constant in the population. Incidence and impairment due to hearing loss appeared consistent in the pandemic year. Once again, Van Hoof et al. were able to demonstrate the negative impact of tinnitus on biopsychosocial quality of life. In particular, they investigated the extent to which tinnitus-specific self-report instruments (TFI and THI) capture aspects of quality of life well and can therefore efficiently replace specific questionnaires on this (SF8 and WHOQOL-BREF). The results show that the Qol subdomain of the TFI does provide sufficient information on quality of life and that the WHOQOL_BREF seems to be the better questionnaire to capture different aspects of quality of life compared to the SF8. Specifically, Wang et al. examined the associations of tinnitus with sleep and anxiety in a cross-sectional study. A total of 45.19% of nearly 400 patients had sleep disturbances, and nearly one-fifth of the population was diagnosed with an anxiety disorder. Female gender, hearing loss, tinnitus exposure, and sleep disturbance proved to be independent predictors of anxiety disorder, with sleep disturbance as a mediator explaining about 28% of the association between anxiety and tinnitus distress. In a prospective study of 100 unilaterally or bilaterally deafened patients or patients with severe asymmetric hearing loss, Olze et al. show a strong improvement of tinnitus-associated complaints after improvement of hearing ability by implant fitting, although the positive effects on tinnitus, anxiety, and depression were less pronounced in the group of the completely deafened. The cooperative research group of the Charité Berlin includes audiological and biological parameters in a longitudinal therapy study on effects of stress, and anxiety on immune activity in tinnitus patients. Negative correlations were found between experienced stress and the number of natural killer cells as well as between anxiety level and the number of regulatory T lymphocytes (Basso et al.). There were no treatment effects in the 40 patients sample, but the number of killer cells as a possible biomarker of tinnitus stress should be further investigated. The work demonstrates the need for individualized, complex therapy via the effects of multiple comorbidities on tinnitus burden and points to the need for multidimensional, holistic therapy in the impact of decompensated tinnitus on various biopsychosocial functions.

Tziridis et al. have previously hypothesized that tinnitus is based on stochastic resonance. In this view, the brain functions to optimize information transfer from sensory system (e.g., the ear) to conscious perception. This mechanism produces tinnitus as a side-product. Here, Tziridis et al. deduce potential sound treatments from this principle and show in a pilot study that this approach may lead to tinnitus reduction. In many sound therapies, the sounds that are used to ameliorate the tinnitus are tailored to the tinnitus pitch. Unfortunately, it is not easy to identify the pitch of tinnitus. Santacruz et al. tested two simple pitch-matching methods. They conclude that a simple multiple-choice pitch-matching method is reliable and has the potential to be broadly applicable in clinical settings. Two studies investigated the audiometric characteristics of patients with tinnitus. Park et al. describe cases of tinnitus in otherwise normal hearing. They describe various subclinical symptoms associated with such cases, where aural fullness and a reduced loudness discomfort levels are common findings. Haider et al. describe a reduced wave-I amplitude in auditory brainstem responses of subjects with tinnitus. They suggest that this can serve as a future audiological biomarker of tinnitus. Spencer et al. studied the feasibility and efficacy of a bimodal stimulation protocol to treat tinnitus. The treatment combines trans-cutaneous electrical stimulation with auditory stimulation. They show the treatment to be feasible and potentially effective in some tinnitus patients. Interestingly, the efficacy is not necessarily limited to patients with somatosensory tinnitus.

### 5.3 Neuroimaging

Riha et al. delved into neurofeedback treatment response patterns for tinnitus. By classifying individuals based on oscillatory trajectories during treatment, they uncovered that a majority were non-responders. However, health-related wellbeing metrics significantly distinguished groups, highlighting the need for individualized approaches. Another study utilized rs-fMRI to distinguish between recent-onset and persistent tinnitus patients. They identified reduced intra-regional brain activity and altered inter-regional connectivity in both groups, emphasizing the necessity for early interventions in recent-onset tinnitus to prevent progression to more persistent and debilitating forms (Du et al.). Through electroencephalography, Lee et al. spotlighted the impact of sudden sensorineural hearing loss on tinnitus generation. Their findings accentuated the role of the “triple brain network” comprising default mode network (DMN), central executive network (CEN), and salience network (SN), suggesting that specific network activations lead to tinnitus onset, while others suppress its manifestation. In their analysis of acute unilateral tinnitus patients with hearing loss, Zhou et al. discovered extensive alterations in 7 major resting-state networks. Their work underscores that multiple network interactions are disrupted early on in tinnitus furthering the understanding neuropathophysiological mechanism of acute tinnitus. Becker et al. linked inflammation to neural activity in tinnitus. They found a significant correlation between inflammation markers, specifically CRP, and decreased (gamma) activity in the orbitofrontal cortex. This suggests that inflammation could intensify tinnitus through the disinhibition of auditory processes, emphasizing the relevance of immune-brain interactions in tinnitus research.

## 6 Summary and perspectives

Bringing a research field forward, and developing it, is a collaborate effort of the researchers in the field. One important part of it, is the development of research tools that are available. The better the research tools, the higher the quality of the research can be. The researchers in the field need to develop their own toolbox of measurement tools, discuss, which tools will be generally accepted, improve them, where needed and agree upon them. In the field of tinnitus, where there is yet no research method that can objectively measure tinnitus, this “tool development task” is of high importance. In this Research Topic, several research teams set themselves the goal to challenge and improve the tinnitus research methods, e.g. for animal research (Fabrizio-Stover et al.), for improving the QOL assessments in humans (Van Hoof et al.), for improving auditory assessments in tinnitus (Park et al.) and the pitch matching methods (Santacruz et al.).

A better understanding of the neuronal mechanisms underlying the conscious perception of tinnitus will also be an important step in the development of better research methods. Despite all the different causes for tinnitus that were discussed in this Research Topics “*Towards an Understanding of Tinnitus Heterogeneity*,” volume 1 and 2, the similarity between all tinnitus subtypes is the conscious perception of a tinnitus sound. This conscious perception is most likely encoded in the neuronal activity in the cortical networks of the affected person. In this Research Topic, we collected a few articles working on a better understanding of these neuronal mechanisms with a variety of different research methods (e.g., fMRI, EEG and MEG) (Du et al.; Lee et al.; Zhou et al.; Becker et al.). The call to improving the research methods on tinnitus is open! - and we will hopefully see much more improvements in tinnitus research methodology during this century.

## Author contributions

CC: Writing – original draft, Writing – review & editing. TK: Writing – original draft, Writing – review & editing. BL: Writing – original draft, Writing – review & editing. AN: Writing – original draft, Writing – review & editing. PN: Writing – original draft, Writing – review & editing. BM: Writing – original draft, Writing – review & editing. PV: Writing – original draft, Writing – review & editing. WS: Writing – original draft, Writing – review & editing.

